# Decrease in healthcare-associated infection rates in preterm infants—longitudinal data from 15 years of nationwide surveillance in Germany

**DOI:** 10.1017/ice.2026.10407

**Published:** 2026-04

**Authors:** Ferenc Darius Ruether, Frank Schwab, Gizem Karadağ, Christine Geffers, Brar Piening

**Affiliations:** 1 Institute of Hygiene and Environmental Medicine, Charité - Universitätsmedizin Berlin, corporate member of Freie Universität Berlin, Berlin, Germany; 2 National Reference Centre for Surveillance of Nosocomial Infections, Berlin, Germany

## Abstract

**Objective:**

Although preterm infants are prone to healthcare-associated infections (HAI), HAI surveillance in neonates is still not widely practiced. In this paper, we present the HAI rates subsequent to nationwide implementation of NEO-KISS, the German national surveillance system for HAI in high-risk neonates. We also report on risk factors for the development of HAI in this population.

**Design:**

Observational study.

**Setting:**

German National Reference Centre for Surveillance of Nosocomial Infections, responsible for the maintenance of NEO-KISS.

**Patients:**

Infants with a birth weight of less than 1500 g.

**Methods:**

NEO-KISS data from the years 2008–2022 was analyzed retrospectively and incidence densities were calculated (in five-year reference periods) for different types of healthcare-associated sepsis (HAS), healthcare-associated pneumonia (HAP), and necrotizing enterocolitis (NEC). Rates were analyzed with Cox-proportional hazard regression models.

**Results:**

A total of 118,214 infants with a birth weight of less than 1500 g from 251 neonatology departments were included. They comprised 15,254 HAS, 1,657 HAP, and 2,786 NEC. The incidence densities of HAS and HAP were 33.3% and 46.7% lower in the admission period 2018-2022 compared to 2008–2012 (2.98 vs. 4.47 HAS, 0.3 vs. 0.56 HAP, per 1,000 patient days). In the multivariable regression analysis, the period of admission remained significant after adjustment for independent risk factors for HAS, HAP, and NEC.

**Conclusions:**

In Germany, the surveillance data for neonates in NEO-KISS between 2008 and 2022 showed a nationwide decrease in incidence densities of HAS, HAP, and NEC. The continuous engagement with NEO-KISS surveillance data may have contributed to this reduction.

## Introduction

Preterm infants, because of their immaturity, are prone to serious infections. Since premature infants spend long stays in hospitals and often require invasive procedures, they are at a particularly high risk of healthcare-associated infections (HAI).^1^ Birth weight and gestational age as well as previous placement of a peripheral or central venous catheter (CVC) have been identified as independent risk factors for healthcare-associated sepsis (HAS).^2,3^ The implementation of a local surveillance system whose reference information for benchmarking allowed timely adjustment of locally implemented infection prevention and control (IPC) measures led to a decrease in HAS incidence density rates of 24% (from 8.3 to 6.4) over a three-year period.^3^ In light of this, it is surprising that in many countries continuous nationwide surveillance of HAI among high-risk neonates is not routine practice and that knowledge of the contribution of long-term surveillance to the prevention of HAI in neonates is still limited. In Germany, though, participation in such a surveillance system has been mandatory for neonatal units seeking classification as high-level (level 1 or level 2) perinatal centers since 2006, and is therefore indirectly linked to financial reimbursement.^4^ This has encouraged the participation of neonatal units in a surveillance system. Because NEO-KISS has in essence been the only established surveillance system in Germany, data has been reported to it nationwide by almost all neonatology departments in Germany. The aim of this paper is to describe the development of HAI rates among high-risk neonates since nationwide implementation of NEO-KISS and to present risk factors in the development of HAI.

## Methods

### Data collection and study population

NEO-KISS is a prospective, patient-based surveillance system for HAI and device use in infants with a birth weight of less than 1500 g. The program was launched in Germany in 2000 by the National Reference Centre for Surveillance of Nosocomial Infections (NRZ). Its focus is HAS, healthcare-associated pneumonia (HAP), and necrotizing enterocolitis (NEC) using modified Centers for Disease Control and Prevention (CDC) definitions.^5–8^ In this context, the term HAI is used throughout the manuscript as an umbrella term encompassing all conditions captured by the NEO-KISS surveillance system, even if NEC is not uniformly attributable to an infectious cause.^9^ Upon enrollment in the program, neonatology departments indicate their level of care as defined by the German joint federal committee. This definition distinguishes four such levels, with level one corresponding to the highest and level four to the lowest. The first two levels include maintenance of a neonatal intensive care unit (NICU).^10^ Case definitions of HAI in NEO-KISS are described in detail in the current NEO-KISS protocols developed by the NRZ.^5,11^ It specifies a HAI be recorded in the database if symptom-onset occurs more than 72 hours after birth/admission. It is considered central venous catheter-associated HAS (CVC-S) or invasive mechanical ventilation-associated HAP (VAP) if the device (CVC, including an umbilical catheter, or a tube) was in place for at least three consecutive calendar days (regardless of the time of placement) either on the day of the first onset of symptoms or on the day before onset of symptoms. Subtypes of HAS are (i) laboratory-confirmed bloodstream infection (BSI) with a blood (or cerebrospinal fluid) culture positive for a recognized pathogen other than coagulase-negative staphylococci (non-CoNS BSI), (ii) BSI with a blood culture positive for CoNS (CoNS BSI), and (iii) clinical HAS without cultured pathogens (clinical HAS). All three subtypes require the presence of at least two clinical symptoms (temperature >38 °C, <36.5 °C, or unstable; tachycardia or bradycardia; apnea; extended recapillarization time; metabolic acidosis; hyperglycemia; or other signs of sepsis) without signs of infection at another site. In addition, a recording of CoNS BSI requires at least one laboratory finding (increased values for C-reactive protein, for interleukin, or for immature and total neutrophil ratio; decreased values for platelets or white blood cells); a recording of clinical HAS requires at least five days of adequate, anti-infective therapy. Overall, the NEO-KISS surveillance system allows reporting of the following categories of HAS: Total HAS events with subtypes CoNS BSI, non-CoNS BSI and clinical HAS; total CVC-S events (as a subset of HAS events) with subtypes CVC-associated CoNS BSI (CVC-CoNS BSI), CVC-associated non-CoNS-BSI (CVC-non-CoNS BSI), and clinical CVC-S.

Criteria for HAP and NEC are defined in the NEO-KISS protocols^11^ and described in Supplementary Table S1. Briefly, diagnosis of HAP requires one radiological finding, deterioration in saturation or gas exchange and at least four clinical criteria. NEC is recorded if at least one radiological sign and two clinical signs are present, or a histological report. Recording of more than one HAI of the same HAI-type per infant is possible if the new episode occurs after an interval of 14 days and if there was an interval that was symptom-free. All neonates admitted to a participating neonatology department with a birth weight below 1500 g are followed up in NEO-KISS until they reach a weight of 1800 g, with the exception of cases which drop out of surveillance because they have been discharged, transferred to another hospital/department, or have died. Key methodological specifications and definitions of NEO-KISS are summarized in Supplementary Table S1 and compared with current CDC/NHSN definitions.

Here, we present an analysis of all infants who were admitted between January 01, 2008, and December 31, 2022, enrolled in NEO-KISS, and subsequently followed up. We did not include data in our analysis from 2006 and 2007, that is two years after financial reimbursement was in place, in order to give new centers an opportunity to successfully navigate the implementation process involved. Primary outcome parameters are device utilization (device days per 100 patient days) and HAI (total number and incidence densities per either 1,000 patient days or 1,000 device days). Secondary outcome was mortality under surveillance (per 100 patient days).

Ethical approval and individual informed consent are not required and institutional review boards were not consulted, firstly because surveillance of HAI and the associated data collection by hospitals is legally mandated by the German Protection Against Infection Act (Infektionsschutzgesetz §23)^12^; and secondly because our analysis and publication of aggregated information for the purpose of improving infection prevention and control is one of our responsibilities, as defined by Robert Koch Institute, in our role as the NRZ for the surveillance of nosocomial infections in Germany on behalf of the Federal Ministry of Health.^13^

### Statistical analysis

Multivariable regression models were used to assess the effect of the development of HAI over time. Three five-year periods of admission (2008–2012, 2013–2017, and 2018–2022) were set up to correspond with the reference period intervals. Cox-proportional hazard regression models with cluster effects were applied which adequately considered the time to event and clusters in a department to calculate hazard ratios (HR) for the first HAS, HAP and NEC of each case.

Each type of HAI (HAS, HAP, NEC) and each subtype of HAS (non-CoNS BSI, CoNS BSI, clinical HAS), including CVC-S and VAP, were analyzed separately. We calculated crude and adjusted hazard ratios with 95% confidence intervals for the period of admission. Separate models were calculated for each type of HAI using variable selection stepwise backward, starting with the full adjusted model. *P* < .05 was considered statistically significant. Data was analyzed using SAS version 9.4 and IBM SPSS Statistics version 29. Figures were created in Microsoft Excel 2019.

## Results

Overall, 251 neonatology departments participated in NEO-KISS during the period January 2008 to December 2022. Of these, the majority were certified as perinatal centers level 1 (*n* = 163, 64.9%) or level 2 (*n* = 67, 26.7%). In the period between 2008 and 2022, the median length of NEO-KISS participation among departments was 15 years (interquartile range (IQR): 11–15). The median number of patients per department was 414 (IQR: 117–695), with a median of 14,244 patient days (IQR: 2,708–25,214) observed (Supplementary Table S2).

**Figure 1.**
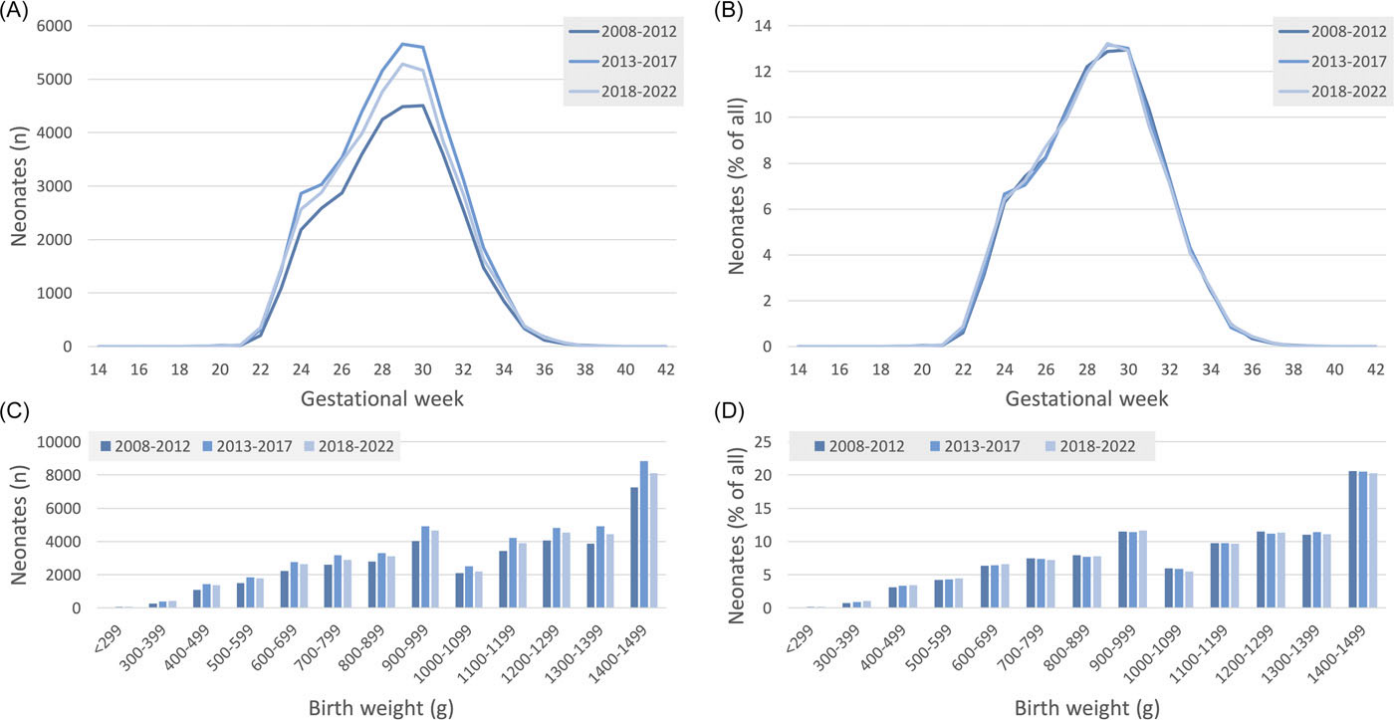
Distribution of gestational age (A: frequency, B: percentage) and birth weight (C: frequency, D: percentage) of neonates during the three surveillance periods, 2008–2012 versus 2013–2017 versus 2018–2022.

A total of 118,214 infants were admitted during the three surveillance periods, comprising 4,206,534 patient days. Almost all infants were admitted to a perinatal center level 1 (92.8%) or level 2 (4.9%). The majority of births were in hospital (92.4%) and by Cesarean section (90.1%). The median birth weight was 1120g (IQR: 830–1355g), with 5,041 (4.3%) neonates weighing less than 500g. The median gestational age was 29 weeks (IQR: 27–31). The distribution of gestational age and birth weight per five-year period is shown in Figure 1A–D.

A total of 61.4% of the infants had at least one CVC day and 43.2% received at least one day of invasive mechanical ventilation. Regarding device utilization rates, infants were equipped with a CVC on an average of 26.2% of patient days and were intubated on an average of 12.8% of patient days. Overall, 96,203 (81.4%) infants were followed up until they reached the surveillance end point of 1800 g body weight, 14,781 (12.5%) were transferred or discharged, and 7,230 (6.1%) died while under surveillance. The median length of surveillance was 31 days (IQR 21–47). Table 1 presents an overview of the respective demographic data. In total, 15,254 HAS, 1,657 HAP and 2,786 NEC were counted in 13 619 (11.5%), 1601 (1.4%), and 2,763 (2.4%) cases, respectively (Table 2).

**Table 1.** Descriptive data for 118,214 neonates analyzed in the study, NEO-KISS 2008–2022, Germany

Parameter	Category	*N*/median	%/IQR
Patients (*N*, %)		118,214	100
Period (*N*, %)	2008–2012	35,120	29.7
	2013–2017	43,075	36.4
	2018–2022	40,019	33.9
Level of care of admitting neonatology department (*N*, %)^1^	Level 1 (highest)	109,678	92.8
	Level 2	5,778	4.9
	Level 3	1,479	1.3
	Level 4 (lowest)	1,279	1.1
Admission (*N*, %)	In-house birth	109,231	92.4
	≤24 h after birth	3,666	3.1
	>24 h after birth	5,316	4.5
Birth weight (median gram, IQR)		1,120	830–1,355
Birth weight category (*N*, %)	1: <500 g	5,041	4.3
	2: 500–999 g	44,111	37.3
	3: 1,000–1,499 g	68,870	58.4
Gestational age (median weeks, IQR)		29	27–31
Sex at birth (*N*, %)	Female	57,495	48.6
CRIB score, recorded for 56,031 (47.4%) patients (median points, IQR)		2	1–5
Delivery mode (*N*, %)	Cesarean section	106,452	90.1
Multiple birth (*N*, %)		38,161	32.6
Patients with CVC (*N*, %)		72,608	61.4
Patients with intubation (*N*, %)		51,121	43.2
End of surveillance (*N*, %)	Death	7,230	6.1
	Over 1800 g	96,203	81.4
	Transfer	14,781	12.5
Patient days and device use			
Total patient days (*N*, %)		4,206,534	
Length of stay until end of surveillance (median days, IQR)		31	21–47
CVC days (*N*, %)		1,101,590	26.2^2^
CVC days per patient (median, IQR)		7	0–13
Intubation days (*N*, %)		540,160	12.8^2^
Intubation days per patient (median, IQR)		0	0–4

CRIB, Clinical risk index for babies; CVC, Central venous catheter; IQR, Interquartile range.

^1^As defined by the German joint federal committee.^10^

^2^Percent of patient days.

**Table 2.** Numbers and percentages of different healthcare-associated infections and affected patients from all 118,214 neonates analyzed in the study, NEO-KISS 2008–2022, Germany

Parameter	Category	*N*/median	%/IQR
HAI-Episodes (*N* = 19,697)			
HAS (*N*, %)	Total	15,254	77.4
HAS by definition (*N*, %)	CoNS BSI	3,771	19.1
	Non-CoNS BSI	3,656	18.6
	Clinical HAS	7,827	39.7
HAS by CVC-association (*N*, %)	CVC-S	7,137	36.2
	CVC-CoNS BSI	2,251	11.4
	CVC-non-CoNS BSI	1670	8.5
	Clinical CVC-S	3,216	16.3
HAP (*N*, %)	Total	1657	8.4
HAP by intubation-association (*N*, %)	VAP	984	5.0
NEC (*N*, %)	total	2,786	14.1
Patients (*N* = 118,214)			
Patients with (≥1) HAS (*N*, %)		13,619	11.5
No. HAS episodes in patients with HAS (*N*, %)	1	12,241	10.4
	2	1,158	1
	3	184	0.2
	4	35	0.03
	5	1	0
Days to first HAS (median, IQR)		15	10–25
Patients with ≥1 CoNS BSI (*N*, %)		3,678	3.1
Patients with ≥1 non-CoNS BSI (*N*, %)		3,541	3.0
Patients with ≥1 clinical HAS (*N*, %)		7,080	6.0
Patients with ≥1 CVC-S (*N*, %)		6,661	5.6
Patients with ≥1 CVC-CoNS BSI (*N*, %)		2,210	1.9
Patients with ≥1 CVC-non-CoNS BSI (*N*, %)		1637	1.4
Patients with ≥1 clinical CVC-S (*N*, %)		3,031	2.6
Days to first CoNS BSI (median, IQR)		14	10–21
Days to first non-CoNS BSI (median, IQR)		16	10–27
Days to first clinical HAS (median, IQR)		18	11–31
Days to first CVC-S (median, IQR)		14	10–22
Patients ≥1 HAP (*N*, %)		1,601	1.4
No. HAP episodes in patients with HAP (*N*, %)	1	1,547	1.3
	2	52	0.04
	3	2	0
Days to first HAP (median, IQR)		20	13–30
Patients ≥1 VAP (*N*, %)		951	0.8
Patients with ≥1 NEC (*N*, %)		2,763	2.3

CVC-S, central venous catheter-associated sepsis; CoNS, coagulase-negative staphylococci; HAS, healthcare-associated sepsis; HAP, healthcare-associated pneumonia; IQR, interquartile range; NEC, necrotizing enterocolitis; VAP, invasive mechanical ventilation-associated pneumonia.

### Healthcare-associated bloodstream infections

With respect to the different categories of HAS, the total number of 15,254 episodes consist of 3,771 CoNS BSI (24.7%) recorded from 3,678 patients, 3,656 non-CoNS BSI (24.0%) from 3,541 patients, and 7,827 clinical HAS (51.3%) from 7,080 patients. More than one HAS was detected in the course of the admission in 1,378 of all affected patients (10.1%). In addition, 7,137 CVC-S episodes (46.8%) were observed in 6,661 infants. Of these, CVC-CoNS BSI accounted for 2,251 episodes (31.5%), which corresponds to 68% more than the number of CoNS BSI in non-CVC-S (*n* = 1,520 / 8,117, 18.7%) (Supplementary Figure S1). Additional descriptive results for the entire observation period are listed in Table 2; results of the individual surveillance periods 2008–2012, 2013–2017, and 2018–2022 are shown in Supplementary Table S3.

**Figure 2.**
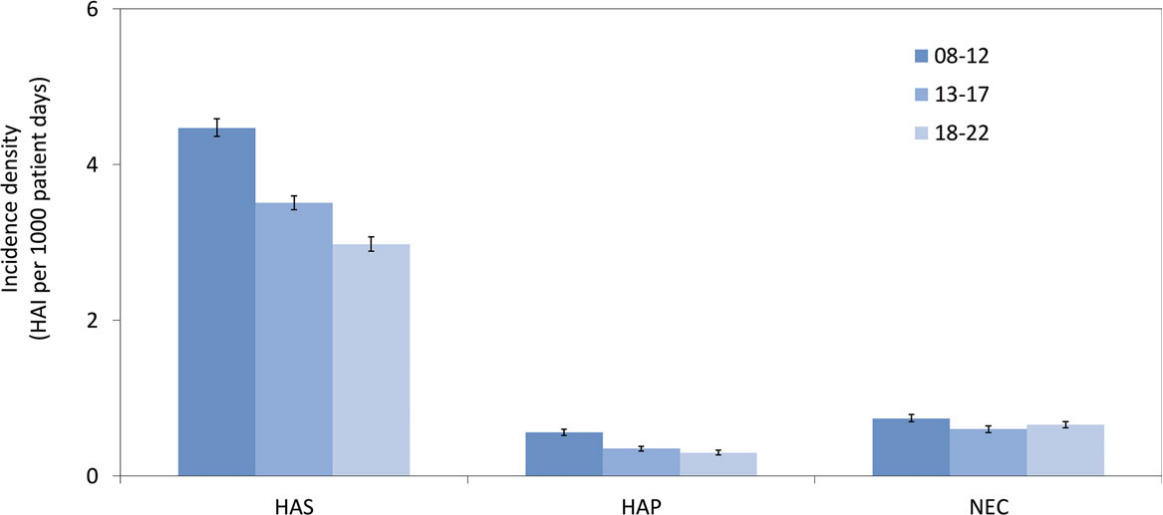
Incidence densities of healthcare-associated sepsis (HAS), healthcare-associated pneumonia (HAP) and necrotizing enterocolitis (NEC) in neonates during the three surveillance periods, 2008–2012 versus 2013–2017 versus 2018–2022. HAI: healthcare-associated infection.

The overall HAS incidence density was 3.63 (per 1,000 patient days), which represents a 33.3% decrease over each of the five-year surveillance periods from 4.47 in 2008–2012 to 2.98 in 2018–2022 (Figure 2).

**Figure 3.**
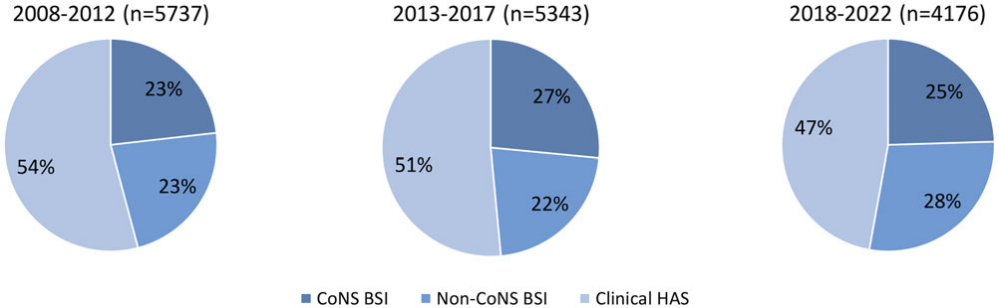
Proportions of different types of healthcare-associated sepsis (HAS) in neonates during the three surveillance periods, 2008–2012 versus 2013–2017 versus 2018–2022. CoNS: coagulase-negative *staphylococci*.

The degree of decline varied among the different subtypes of HAS. The incidence densities of clinical HAS, CoNS BSI, and non-CoNS BSI decreased from 2.42 to 1.41 (–41.7%), from 1.04 to 0.73 (–29.8%) and from 1.01 to 0.84 (–16.8%), respectively, (Supplementary Table S3). It is noteworthy that from 2013–2017 to 2018–2022 non-CoNS BSI rates increased by 9% from 0.77 to 0.84 with an associated higher percentage of non-CoNS BSI (relative to all HAS) compared to the earlier periods (Figure 3).

Although CVC days per patient did not decline during the observation period (Supplementary Table S3, Supplementary Figure S2), the CVC-S rate decreased by 28.7% from 7.74 to 5.52. In terms of birth weight and gestational age of infants, incidence densities of all types of HAS decreased significantly as birth weight and gestational age increased (Supplementary Table S5).

### Healthcare-associated pneumonia and necrotizing enterocolitis

Regarding HAP, 984 of 1,657 episodes (59.4%) were assessed as VAP (Table 2) a trend that decreased slightly over the surveillance periods (Supplementary Table S3). The overall incidence density was 0.39, which declined by 46.7% from 0.56 during 2008–2012 to 0.3 in 2018–2022 (Figure 2, Supplementary Table S4). Rates of VAP fell from 2.22 to 1.44 (–35.1%) (Supplementary Figure S2), while the intubation rates decreased by 24.5% from 14.7 to 11.1 (Supplementary Table S3). In contrast, NEC incidence densities did not show a clear trend over time (Figure 2). However, both HAP and NEC rates decreased as birth weight and gestational age increased (Supplementary Table S5).

### Results of regression analysis

**Figure 4.**
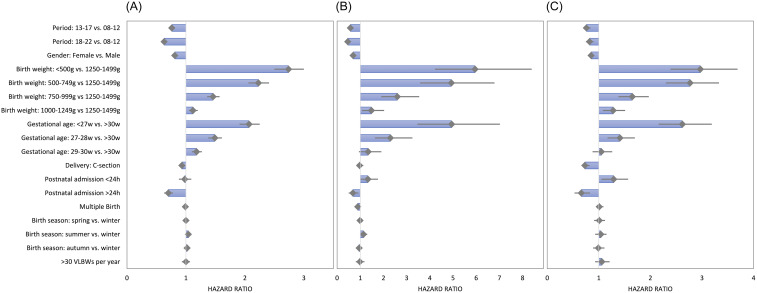
Results of multivariable cox-proportional hazard regression analysis for the occurrence of healthcare-associated sepsis (A), healthcare-associated pneumonia (B) and necrotizing enterocolitis (C) in neonates. VLBW: very low birth weight (<1500 g) infants.

Multivariate regression analysis revealed significant risk reduction for (the first) HAS during the second (2013–2017) and third (2018–2022) period of admission compared to 2008–2012 with hazard ratios (HR) of 0.76 (95% confidence interval (CI): 0.73–0.79) and 0.63 (95% CI: 0.61–0.66) (Supplementary Table S6). A reduced risk was also observed for females (HR 0.81, 95% CI 0.79–0.84), Cesarean births (HR 0.93, 95% CI 0.88–0.98), and admission > 24 h after birth (HR 0.7, 95% CI: 0.63–0.78). An increased risk was found for decreasing birth weight (HR 1000–1249 g vs. 1250–1499 g: 1.12, 95% CI: 1.05–1.2; HR < 500 g vs. 1250–1499 g: 2.74, 95% CI: 2.5–3) and younger gestational age (HR 29–30 weeks vs. >30 weeks: 1.18, 95% CI: 1.1–1.27; HR <27 weeks vs. >30 weeks: 2.07, 95% CI 1.91–2.25) (Figure 4A).

While CoNS BSI, clinical HAS, and CVC-S yielded similar results, the risk of non-CoNS BSI increased again slightly from 2013–2017 to 2018–2022 (Supplementary Table S7).

Admission during a later surveillance period also reduced the risk of HAP. However, no decrease was observed for NEC. HAP was less frequent in cases of multiple birth (HR 0.89, 95% CI: 0.81–0.98) and NEC occurred less often after Cesarean sections (HR 0.73, 95% CI 0.66–0.82) (Figure 4B–C).

## Discussion

With almost 120,000 preterm infants and over four million patient days, this paper describes one of the largest cohorts of high-risk neonates ever prospectively monitored for surveillance purposes. When compared with the total number of live births in Germany between 2008–2022 (*n* = 132,138, including two years with estimated numbers) with a birth weight less than 1500 g,^14,15^ the reported data encompasses more than 80% of all infants born in Germany during this time period with a birth weight below 1500 g. Moreover, the median length of participation in NEO-KISS of 15 years per department indicates that most departments participated continuously. Therefore, the sample can be considered a comprehensive representation of nationwide data and demonstrates the feasibility of continuous HAI surveillance in neonates in a high-income EU country.

The observed incidence of HAS of 11.5% (13,619/118,214) for the years 2008–2022 was lower than the sepsis incidence of 17.7% identified using ICD-10 codes among infants with a birth weight of <1500 g, as previously reported in a population-wide analysis from Germany.^16^ However, in contrast to NEO-KISS, the authors of that study did not exclude cases of early-onset sepsis.

A strength of NEO-KISS is that not only are laboratory-confirmed cases of HAS registered, but blood culture-negative, clinically diagnosed HAS are as well. Although the clinical decisions associated with this type of HAS tend to be more subjective—and therefore a recording of clinical HAS as well—more than 50% of all observed HAS occurred with negative cultures. Since a percentage of 90% of blood culture-negative cases were found in other studies for late-onset sepsis,^17^ monitoring for clinical sepsis is an essential part of the surveillance of high-risk neonates.

The observation of a higher percentage of CoNS BSI and a smaller percentage of clinical HAS in CVC-S compared to non-CVC-S is in line with other studies that detected CoNS in 75% of central line-associated BSI vs. 15–68% in other BSI.^18^ Of note, in the study by Jansen *et al*., a HAS was defined by at least one positive blood culture and could not be diagnosed based on clinical judgment alone.

The overall decrease in the HAI incidence density (Figure 2) is well aligned with the global decrease in sepsis incidence across all patient groups since 1990.^19^ This may in general reflect improved medical care. Given the fact that CoNS BSI are more likely to be associated with contamination,^20^ the greater reduction in CoNS BSI incidence density compared with non-CoNS BSI incidence density indicates that IPC measures are among the improvements that have been made, including improved CVC management, early enteral and human milk feeding, use of closed systems for blood sampling, strict adherence to sterile techniques, and, in some settings, the use of probiotics.^21^ Interestingly, no relevant reduction in HAI rates were observed in other longitudinal cohorts that included preterm infants.^18,22^ However, these cohorts were not placed under surveillance or did not originate from a single center.

This highlights the importance of a surveillance network like NEO-KISS, which allows for periodic feedback and benchmarking. We can therefore assume that engaging with the surveillance data, together with other IPC measures such as prevention bundles and better training,^23,24^ has contributed to a reduction in the risk of CVC-S. A similar observation was made in a single center study from Nova Scotia that analyzed data from 16 years of surveillance of pediatric patients. A remarkable 84% reduction in central line-associated BSI rates was observed and associated with a simultaneous hand hygiene campaign.^25^

Comparable results were found for HAP. In relative terms, its decline over the years has been even greater than that of HAS (47% vs. 33%). Moreover, the rate of VAP decreased by 35%. In contrast to CVC, there was a remarkable drop in the intubation rate. This is most likely related to an increased use of noninvasive techniques like continuous positive airway pressure via supraglottic tube, binasal prongs, and especially high-flow nasal cannula intended to prevent morbidities related to invasive ventilation such as VAP in neonates.^26^ Furthermore, the reduction of HAP rates can probably be attributed to an increasing effort to implement bundles to prevent HAP.^27^

NEC incidence densities fluctuated over the surveillance periods and did not show a clear trend. This might reflect the complex, multifactor etiology of this partially endogenous syndrome.^28^ Therefore, the link to causative health care processes and the effect of IPC interventions is difficult to assess for NEC. However, we consider surveillance of NEC a beneficial tool for identifying clusters nonetheless.^29^

A later period of admission was independently associated with lower risk of HAS and HAP in the multivariate regression analysis. Moreover, a lower gestational week and birth weight were confirmed as risk factors for HAI, which is in line with the results from large cohort studies.^30,31^

The substantial reduction in the risk of NEC after a Cesarean section is also noteworthy. Although similar results have been reported in the past, there have also been reports showing no difference or even a higher risk following a Cesarean section.^32^ This indicates the need for further investigation of this question.

The following limitations need to be considered.

First, manual surveillance always depends on the expertise and accuracy of the person responsible for carrying out the surveillance. Therefore, the NRZ organizes yearly events to instruct local IPC staff in the validated clinical criteria in the NEO-KISS protocol. Participating hospitals are required to guarantee that the recording of data is performed only by IPC staff that have had this training. Second, in 2017, during the observation period, two relevant changes to the NEO-KISS protocol were introduced and implemented. These were i) a cutoff period of 14 days, in reference to which a second infection in the same organ or with the same bacterial species cannot be diagnosed until at least 14 days after the first infection, and ii) by definition, “device-association” applies only if the device has been in place for at least three days either on the day of the first onset of symptoms or on the day before onset of symptoms. Third, device days could not be incorporated into the analysis, as the available data do not distinguish between a device preceding infection and a device occurring as a consequence of the infection. Fourth, since blood culture sampling frequency is not recorded in NEO-KISS, the changes in HAS rates could ultimately have been the result of changes in blood culture sampling policies. Fifth, comparability of NEO-KISS with surveillance systems from other countries is limited, but our results strongly support the goal of initiatives that aim at introducing internationally harmonized surveillance systems for nosocomial infections in high-risk neonates similar to the NeoIPC Surveillance.^33^

Here, for the first time, data is presented from a nationwide surveillance system for HAI in high-risk neonates which has been maintained continuously in Germany for more than two decades. Evaluation based on surveillance periods shows a continuous reduction in incidence densities of HAS and HAP over time. In conclusion, we interpret this decrease as a sign of improved treatment, which includes improved IPC measures and the ongoing surveillance of HAI. The feasibility and success of a nationwide surveillance system for preterm infants should encourage wider adoption of methods like NEO-KISS.

## Supporting information

Ruether et al. supplementary material Ruether et al. supplementary material

## Data Availability

Not applicable, because all data were collected as part of the surveillance of (HAI) conducted in accordance with the German Protection Against Infection Act.
