# Supplementary material for: Decrease in healthcare-associated infection rates in preterm infants—longitudinal data from 15 years of nationwide surveillance in Germany
Source: Infect Control Hosp Epidemiol. 2026 Mar 5;47(4):394–401. doi: 10.1017/ice.2026.10407 (PMC13216796; doi:10.1017/ice.2026.10407)
Supplement: Ruether et al. supplementary material [file S0899823X26104073sup001.docx]

**Supplementary Material**

Decrease in healthcare-associated infection rates in preterm infants – longitudinal data from 15 years of nationwide surveillance in Germany

**Supplementary Table S1:**
[Side-by-side comparison of the current NEO-KISS and the CDC/NHSN definitions](#_Supplementary__Table)

**Supplementary Table S2:**
[Descriptive data of 251 neonatology departments analysed in the study, NEO-KISS 2008-2022, Germany.](#_Supplementary_Table_S2:)

**Supplementary Figure S1:**[Proportions of different types of healthcare-associated sepsis (HAS) during the observation period, 2008-2022.](#_s)

**Supplementary Table S3:**[Descriptive data of neonates and acquired healthcare-associated infections during the three surveillance periods, 2008-2012 vs. 2013-2017 vs. 2018-2022](#_Supplementary_Table_S3:)

**Supplementary Table S4:**[Incidence densities with 95% confidence intervals of healthcare-associated infections in neonates during the overall observation period and the three surveillance periods, 2008-2012 vs. 2013-2017 vs. 2018-2022](#_Supplementary_Table_S4:)

**Supplementary Figure S2:**[Incidence densities of device-associated healthcare-associated sepsis and pneumonia in neonates during the different surveillance periods, 2008-2012 vs. 2013-2017 vs. 2018-2022.](#_T)

**Supplementary Table S5:**[Descriptive data of neonates and acquired healthcare-associated infections for different gestational ages and birth weights](#_Supplementary_Table_S5:)

**Supplementary Table S6:**[Adjusted Hazard ratios for the different types of healthcare-associated infections in neonates calculated for the three surveillance periods by multivariable Cox-proportional hazard regression models.](#_Supplementary_Table_S6:)

**Supplementary Table S7:**[Results of multivariable Cox-proportional Hazard regression analysis for the subtypes of healthcare-associated sepsis in neonates (adjusted full model)](#_Supplementary_Table_S7:)

# **Supplementary Table S1: Side-by-side comparison of the current NEO-KISS and the CDC/NHSN definitions**

|  | **NEO-KISS**  **(based on protocol version 06/2020)^1^** | **CDC/NHSN**  **(based on protocol version 01/2025)^2^** |
| --- | --- | --- |
| General / concept | - Specifically for VLBW infants (<1500g), tracking from admission until discharge, death, or reaching 1800g. - Patient-based (only including VLBW infants (<1500g)) - Voluntary hospital participation with national benchmarking, but participation linked with financial reimbursement | - Covers a broader range of patient groups, with neonatal data being a part of its overall surveillance (defined as patients ≤1 year old). The separate Late-Onset Sepsis (LOS) module^3^ includes infants only with a birth weight between 401 and 1500 grams AND Day of Life (DOL) 4-120 (DOL 1 = Date of Birth)) - Unit-based - Voluntary hospital participation with national benchmarking. |
| Definition of healthcare-associated infections (HAI) | The 72h interval after birth applies; an infection occurring earlier is generally not considered healthcare-associated.  A neonatal infection that has occurred as a result of passage through the birth canal is defined as healthcare-associated, whereas transplacentally acquired infections are not categorised as healthcare-associated. | An infection is considered a HAI if the date of event of the NHSN site-specific infection criterion occurs on or after the 3rd calendar day of admission to an inpatient location where day of admission is calendar day 1.  The LOS module defines a minimum DOL of 4 for inclusion. |
| Blocking period / repeat infection timeframe | A change of pathogen alone is not sufficient to record a new infection after an already recorded infection. For the recording of a new infection of the same organ system, there is a blocking period of 14 days from the onset of the previous and a clinically free interval is also required before the new infection. | CDC/NHSN defines a similar 14-day repeat infection timeframe. The requirement of a clinically free interval is not specified. |
| Types of recorded healthcare-associated sepsis (HAS) / bloodstream infection (BSI) | NEO-KISS distinguishes between three different primary forms of HAS:  • Clinical HAS (without pathogen detection),  • Microbiologically confirmed BSI with pathogen detection (but no CoNS) and  • Microbiologically confirmed BSI with coagulase-negative staphylococci (CoNS) as the sole pathogen  Requirement: BSI not secondary to an infection at another body site | CDC/NHSN distinguishes between three different primary forms of BSI:  • Laboratory-Confirmed BSI (LCBI) 1  • LCBI 2  • LCBI 3  The LOS module distinguishes between two different primary forms of BSI: • Neonatal LCBI 1  • Neonatal LCBI 2  Requirement: BSI not secondary to an infection at another body site |
| Clinical HAS | ALL of the following criteria:  1. attending physician starts appropriate antimicrobial therapy for sepsis for at least 5 days  2. NO pathogen detection in the blood culture or not tested  3. NO obvious infection elsewhere  AND two of the following criteria (without any other recognisable cause)  ▪ Fever (>38° C) or temperature instability (frequent readjustment of the incubator) or hypothermia (<36.5° C)  ▪ Unexplained metabolic acidosis (BE < -10 mval/l)  ▪ Tachycardia (> 200/min) or new/increased bradycardia (<80/min)  ▪ New onset of hyperglycaemia (>140mg/dl)  ▪ Recapillarisation time (RKZ) >2s  ▪ Other signs of sepsis (skin colour (only if RCC is not used), laboratory signs (CRP, interleukin), increased oxygen demand (intubation), unstable, apathy)  ▪ New or increased apnoea(s) (>20s)  A single detection of CoNS in the blood culture does not necessarily rule out the diagnosis of clinical HAS. Clinical HAS can also be diagnosed if CoNS have grown once in the blood culture, this is evaluated as contamination of the blood culture, but the other criteria of CoNS BSI are not fulfilled and those of clinical HAS are fulfilled. | Not defined in the current CDC/NHSN protocol |
| Non-CoNS BSI (NEO-KISS) compared to LCBI 1 and NLCBI 1 (CDC/NHSN) | Pathogen isolated from blood or cerebrospinal fluid that is not a CoNS  (Pathogen must not be related to infection elsewhere)  AND two of the following criteria  ▪ Fever (>38° C) or temperature instability (frequent readjustment of the incubator) or hypothermia (<36.5° C)  ▪ Unexplained metabolic acidosis (BE < -10 mval/l)  ▪ Tachycardia (> 200/min) or new/increased bradycardia (<80/min)  ▪ New onset of hyperglycaemia (>140mg/dl)  ▪ Recapillarisation time (RKZ) >2s  ▪ Other signs of sepsis (skin colour (only if RCC is not used), laboratory signs (CRP, interleukin), increased oxygen demand (intubation), unstable AZ, apathy)  ▪ New or increased apnoea(s) (>20s) | LCBI 1:  Patient of any age has a recognized bacterial or fungal pathogen, not included on the NHSN common commensal list:  1. Identified from one or more blood specimens obtained by a culture  OR  2. Identified to the genus or species level by non-culture based microbiologic testing  (NCT) methods (for example, T2 Magnetic Resonance [T2MR] or next-generation sequencing [NGS]).  AND  Organism(s) identified in blood is not related to an infection at another site  NLCBI 1:  One or more positive blood specimens with a recognized pathogen specifically a bacterial or fungal organism which is NOT a common commensal that can be accessed via the NHSN Terminology Browser. |
| CoNS BSI (NEO-KISS) compared to LCBI 2, LCBI 3 and NLCBI 2 (CDC/NHSN) | CoNS is the only pathogen isolated from blood  AND ONE of the following laboratory parameters (without any other recognisable cause)  ▪ CRP >2.0mg/dl or interleukin  ▪ I/T ratio >0.2 (immature granulocytes / total granulocytes)  ▪ Thrombocytes < 100/nl  ▪ Leukocytes < 5/nl (without erythroblasts)  AND two of the following criteria (without any other recognisable cause)  ▪ Fever (>38° C) or temperature instability (frequent readjustment of the incubator) or hypothermia (<36.5° C)  ▪ Unexplained metabolic acidosis (BE < -10 mval/l)  ▪ Tachycardia (> 200/min) or new/increased bradycardia (<80/min)  ▪ New onset of hyperglycaemia (>140mg/dl)  ▪ Recapillarisation time >2s  ▪ Other signs of sepsis (skin colour (only if RCC is not used), increased oxygen requirement  (intubation), unstable AZ, apathy)  ▪ New or increased apnoea(s) (>20s) | LCBI 2:  Patient of any age has at least one of the following signs or symptoms: fever (>38.0°C), chills, or hypotension  AND  Organism(s) identified in blood is not related to an infection at another site  AND  The same NHSN common commensal is identified by culture from two or more blood specimens collected on separate occasions  LCBI 3:  Patient ≤ 1 year of age has at least one of the following signs or symptoms:  ▪ fever (>38.0°C), hypothermia (<36.0°C), apnea, or bradycardia  AND  Organism(s) identified in blood is not related to an infection at another site  AND  The same NHSN common commensal is identified by a culture from two or more blood specimens collected on separate occasions  NLCBI 2:  One or more positive blood specimens with a common commensal specifically, a  bacterial organism which is a common commensal on the NHSN Organism List that can be accessed via the NHSN Terminology Browser. In addition, a new intravenous antimicrobial agent must be initiated during the LOS/MEN window period on or after DOL 4 AND continued for at least 5 calendar days. |
| Mucosal barrier injury laboratory-confirmed bloodstream infection (MBI-LCBI) | Not defined in NEO-KISS | Once an LCBI determination is made, proceed to the MBI-LCBI definitions, and determine if  the corresponding MBI-LCBI criteria are also met |
| Central venous catheter-associated sepsis (CVC-S) | HAS is associated with CVC if a central vascular access was in place for at least the 3rd day on the day with the first symptoms of infection (=day of infection) or the day before.  A CVC is considered to have been in place for at least 3 days if it has been present on three consecutive calendar days, with the day of insertion counted as day 1, regardless of the time of placement. Accordingly, an event is eligible if it occurs on day 3 or later after device insertion. This definition does not require the device to have been in place for more than 48 hours. | A laboratory confirmed bloodstream infection where an eligible BSI organism is identified, and an eligible central line (CL) is present on the LCBI day of event or the day before.  Eligible CL: has been in place for more than two consecutive calendar days (on or after CL Day 3), following the first access of the CL, in an inpatient location, during the current admission. Such lines remain eligible for CLABSI events until the day after removal from the body or patient discharge, whichever comes first. |
| Pneumonia | ONE radiological finding  ▪ New or progressive infiltrate  ▪ Shading  ▪ Fluid in the interlobar or pleural space  AND deterioration in gas exchange* or drop in saturation  *increase in FiO2 requirement >10% within 24h or start of mechanical ventilation  AND FOUR of the following criteria  ▪ Newly occurring or increased  bradycardia (< 80/min) or  new/increased tachycardia  (>200/min)  ▪ New/increased tachypnoea  (>60/min) or new/increased apnoea (> 20 s)  ▪ purulent tracheal secretion  ▪ Germ from tracheal secretion  ▪ New/increased dyspnoea (retractions, nasal flaring, moaning)  ▪ Temperature instability/fever/hypothermia  ▪ Increased respiratory secretion  (increased aspiration)  ▪ CRP > 2.0 mg/dl or interleukin*  ▪ I/T ratio > 0 | PNU1:  Imaging Test Evidence  TWO or more serial chest imaging test results with at least ONE of the following:  New and persistent or progressive and persistent  ▪ Infiltrate  ▪ Consolidation  ▪ Cavitation  ▪ Pneumatoceles, in infants ≤1 year old  AND for infants ≤ 1 year old signs/symptoms criteria:  ▪ Worsening gas exchange (for example, O2 desaturations [for example, pulse oximetry < 94%], increased oxygen requirements, or increased ventilator demand)  AND at least three of the following (from separate bullets):  ▪ Temperature instability  ▪ Leukopenia (≤ 4000 WBC/mm3) or leukocytosis (≥ 15,000 WBC/mm3) and left shift (≥ 10% band forms)  ▪ New onset of purulent sputum or change in character of sputum, or increased respiratory secretions, or increased suctioning requirements  ▪ Apnea, tachypnea, nasal flaring with retraction of chest wall, or nasal flaring with grunting  ▪ Wheezing, rales, or rhonchi  ▪ Cough  ▪ Bradycardia (< 100 beats/min) or tachycardia (> 170 beats/min)  (Definitions of PNU2 and PNU3 not relevant for comparison with NEO-KISS definitions) |
| Invasive mechanical ventilation-associated pneumonia (VAP) | Pneumonia where invasive ventilation via an endotracheal tube (not "revenge tube") has taken place for at least the 3rd day on the day with the first symptoms of the infection (=day of infection) or on the day before.  Of note, in NEO-KISS, non-invasive ventilation (NIV)-associated events (via high-flow or CPAP) are also recorded, but not reported here. If a patient fulfils both the definition of ventilator-associated and NIV-associated, only the more invasive procedure is considered and the pneumonia is assessed as VAP. | Pneumonia where the patient is on mechanical ventilation for > 2 consecutive calendar days on the date of event, with day of ventilator placement being Day 1  AND the ventilator was in place on the date of event or the day before.  Ventilation and lung expansion devices that deliver positive pressure to the airway (for example, CPAP, BiPAP, Bi-level, IPPB, and PEEP) via non-invasive means (for example, nasal prongs, nasal mask, full face mask, total mask, etc.) are not considered ventilators unless positive pressure is delivered via an artificial airway (oral/nasal endotracheal or tracheostomy tube). |
| Necrotizing enterocolitis (NEC) | ONE of the following radiological* signs  ▪ Pneumoperitoneum  ▪ Pneumatosis intestinalis (gas bubbles in the intestinal wall)  ▪ Unchanged standing loops of small intestine  AND TWO of the following criteria (without any other cause)  ▪ Vomiting  ▪ Food ("stomach") residues  ▪ distended abdomen  ▪ Flank erythema  ▪ Repeated microscopic (haemoccult) or macroscopic blood in the stool  OR  Diagnosis by histological examination of the surgical specimen | ONE imaging test finding which if equivocal is supported by clinical correlation (specifically, physician documentation or physician designee of antimicrobial treatment for NEC):  ▪ Pneumatosis intestinalis  ▪ Portal venous gas (Hepatobiliary gas)  ▪ Pneumoperitoneum  AND ONE clinical sign:  ▪ bilious aspirate (bilious aspirate from a transpyloric feeding tube should be excluded)  ▪ vomiting  ▪ abdominal distention  ▪ occult or gross blood in stools (with no rectal fissure)  OR  Surgical NEC: Infant has at least one of the following surgical findings:  ▪ surgical evidence of extensive bowel necrosis (>2 cm of bowel affected)  ▪ surgical evidence of pneumatosis intestinalis with or without intestinal perforation |
| References:  1: <https://www.nrz-hygiene.de/files/Protokolle/Englisch/NEO-KISS/NEO-KISS%20Protocol%20English.pdf>  2: <https://www.cdc.gov/nhsn/acute-care-hospital/index.html>  3: <https://www.cdc.gov/nhsn/pdfs/neonatal/losmen/los-men-protocol-508.pdf> | | |

# **Supplementary Table S2: Descriptive data of 251 neonatology departments analysed in the study, NEO-KISS 2008-2022, Germany.**

| **Parameter** | **Category** | **N / median** | **% / IQR** |
| --- | --- | --- | --- |
| Departments |  | 251 | 100 |
| Level of neonatal care* | Level 1 (highest) | 163 | 64.9 |
|  | Level 2 | 67 | 26.7 |
|  | Level 3 | 15 | 6.0 |
|  | Level 4 (lowest) | 6 | 2.4 |
| Size of department (beds) |  | 16 | 12-24 |
| Size of hospital (beds) |  | 579 | 401-911 |
| Years analysed |  | 15 | 11-15 |
| Patients |  | 414 | 117-695 |
| Patient days |  | 14244 | 2708-25214 |
| Patients per year |  | 33 | 15-53 |
| *as defined by the German joint federal committee. IQR: interquartile range. | | | |

# s

**
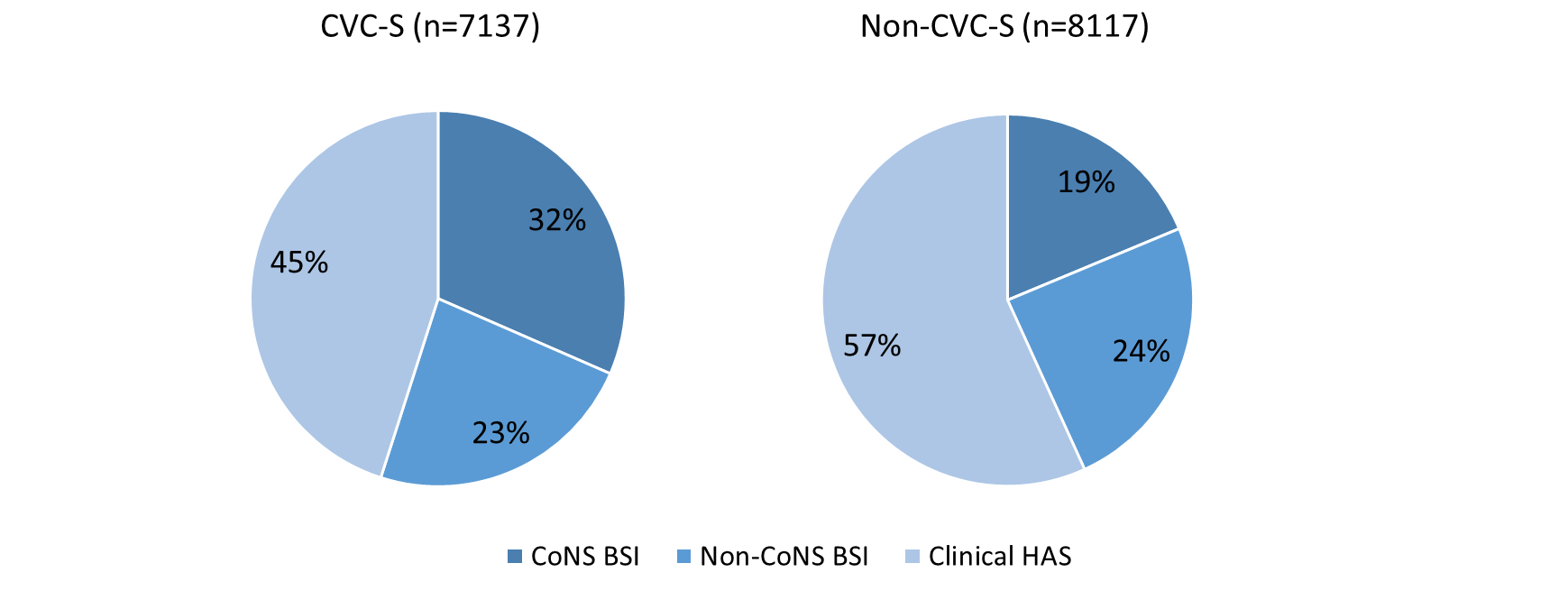
**

**Supplementary Figure S1: Proportions of different types of healthcare-associated sepsis (HAS) during the observation period, 2008-2022.**

BSI: bloodstream infection; CoNS: coagulase-negative staphylococci; CVC-S: central venous catheter-associated sepsis

# **Supplementary Table S3: Descriptive data of neonates and acquired healthcare-associated infections during the three surveillance periods, 2008-2012 vs. 2013-2017 vs. 2018-2022**

| **Parameter** | **Category** | **2008-2012**  **N (%) / median (IQR)** | **2013-2017**  **N (%) / median (IQR)** | **2018-2022**  **N (%) / median (IQR)** | **p-value** |
| --- | --- | --- | --- | --- | --- |
| **Denominator data and device use** | | | | | |
| Patients |  | 35120 (100%) | 43075 (100%) | 40019 (100%) |  |
| Total patient days |  | 1282532 (100%) | 1522573 (100%) | 1401429 (100%) |  |
| Length of stay (patient days) until end of surveillance |  | 32 (22-48) | 31 (21-47) | 30 (20-47) | <0.001 |
| Patients with CVC |  | 20611 (58.7% of patients) | 26380 (61.2% of patients) | 25617 (64% of patients) | <0.001 |
| CVC days |  | 327000 (25.5% of patient days) | 398583 (26.2% of patient days) | 376007 (26.8% of patient days) |  |
| CVC days per patient |  | 6 (0-14) | 6 (0-13) | 7 (0-13) | <0.001 |
| Patients with intubation (Tube) |  | 16760 (47.7% of patients) | 18806 (43.7% of patients) | 15555 (38.9% of patients) | <0.001 |
| Intubation days |  | 188854 (14.7% of patient days) | 195404 (12.8% of patient days) | 155902 (11.1% of patient days) |  |
| Intubation days per patient |  | 0 (0-5) | 0 (0-4) | 0 (0-3) | <0.001 |
| **Episodes** | | | | | |
| **HAI (all)** | total | 7402 (100%) | 6781 (100%) | 5514 (100%) |  |
| **HAS** | total | 5735 (77.5%) | 5343 (78.8%) | 4176 (75.7%) |  |
| HAS by definition | CoNS BSI | 1330 (18.0%) | 1417 (20.9%) | 1024 (18.6%) |  |
|  | Non-CoNS BSI | 1300 (17.6%) | 1174 (17.3%) | 1182 (21.4%) |  |
|  | Clinical HAS | 3105 (41.9%) | 2752 (40.6%) | 1970 (35.7%) |  |
| HAS by central line-association | CVC-S | 2530 (34.2%) | 2532 (37.3%) | 2075 (37.6%) |  |
|  | CVC-CoNS BSI | 721 (9.7%) | 862 (12.7%) | 668 (12.1%) |  |
|  | CVC-non-CoNS BSI | 568 (7.7%) | 532 (7.8%) | 570 (10.3%) |  |
|  | Clinical CVC-S | 1241 (16.8%) | 1138 (16.8%) | 837 (15.2%) |  |
| **HAP** | total | 712 (9.6%) | 531 (7.8%) | 414 (7.5%) |  |
| HAP by intubation-association | VAP | 420 (5.7%) | 339 (5.0%) | 225 (4.1%) |  |
| **NEC** | total | 955 (12.9%) | 907 (13.4%) | 924 (16.8%) |  |
| **Patients** | | | | | |
| ≥1 HAS |  | 4988 (14.2%) | 4799 (11.1%) | 3832 (9.6%) | <0.001 |
| ≥1 CoNS BSI |  | 1301 (3.7%) | 1381 (3.2%) | 996 (2.5%) | <0.001 |
| ≥1 non-CoNS BSI |  | 1247 (3.6%) | 1138 (2.6%) | 1156 (2.9%) | <0.001 |
| ≥1 clinical HAS |  | 2752 (7.8%) | 2508 (5.8%) | 1820 (4.5%) | <0.001 |
| ≥1 CVC-S |  | 2313 (6.6%) | 2376 (5.5%) | 1972 (4.9%) | <0.001 |
| ≥1 HAP |  | 675 (1.9%) | 515 (1.2%) | 411 (1.0%) | <0.001 |
| ≥1 VAP |  | 401 (1.1%) | 327 (0.8%) | 223 (0.6%) | <0.001 |
| ≥1 NEC |  | 945 (2.7%) | 900 (2.1%) | 918 (2.3%) | <0.001 |
| End of surveillance | Death | 2214 (6.3%) | 2578 (6.0%) | 2438 (6.1%) | 0.174 |
|  | Transfer | 4225 (12.0%) | 5251 (12.2%) | 5305 (13.3%) |  |
|  | 1800g | 28681 (81.7%) | 35246 (81.8%) | 32276 (80.6%) |  |
| BSI: bloodstream infection; CoNS: coagulase-negative staphylococci; CVC: central venous catheter; CVC-S: central venous catheter associated sepsis; HAI: healthcare-associated infection; HAP: healthcare-associated pneumonia; HAS: healthcare-associated sepsis; IQR: interquartile range; NEC: necrotising enterocolitis; VAP: invasive mechanical ventilation-associated pneumonia | | | | | |

# **Supplementary Table S4: Incidence densities with 95% confidence intervals of healthcare-associated infections in neonates during the overall observation period and the three surveillance periods, 2008-2012 vs. 2013-2017 vs. 2018-2022**

|  | **2008-2022** | **2008-2012** | **2013-2017** | **2018-2022** |
| --- | --- | --- | --- | --- |
| Incidence densities per 1000 patient days (95%CI) |  |  |  |  |
| HAS | 3.63 (3.57-3.68) | 4.47 (4.36-4.59) | 3.51 (3.42-3.6) | 2.98 (2.89-3.07) |
| Non-CoNS BSI | 0.87 (0.84-0.9) | 1.01 (0.96-1.07) | 0.77 (0.73-0.82) | 0.84 (0.8-0.89) |
| CoNS BSI | 0.9 (0.87-0.93) | 1.04 (0.98-1.09) | 0.93 (0.88-0.98) | 0.73 (0.69-0.78) |
| Clinical HAS | 1.86 (1.82-1.9) | 2.42 (2.34-2.51) | 1.81 (1.74-1.88) | 1.41 (1.34-1.47) |
| HAP | 0.39 (0.38-0.41) | 0.56 (0.52-0.6) | 0.35 (0.32-0.38) | 0.3 (0.27-0.33) |
| NEC | 0.66 (0.64-0.69) | 0.74 (0.7-0.79) | 0.6 (0.56-0.64) | 0.66 (0.62-0.7) |
| Device associated Incidence densities per 1000 device days (95%CI) |  |  |  |  |
| CVC-S | 6.48 (6.33-6.63) | 7.74 (7.44-8.04) | 6.35 (6.11-6.6) | 5.52 (5.28-5.76) |
| CVC-non-CoNS BSI | 1.52 (1.44-1.59) | 1.74 (1.6-1.89) | 1.33 (1.22-1.45) | 1.52 (1.39-1.65) |
| CVC-CoNS BSI | 2.04 (1.96-2.13) | 2.2 (2.05-2.37) | 2.16 (2.02-2.31) | 1.78 (1.64-1.92) |
| Clinical CVC-S | 2.92 (2.82-3.02) | 3.8 (3.59-4.01) | 2.86 (2.69-3.03) | 2.23 (2.08-2.38) |
| VAP | 1.82 (1.71-1.94) | 2.22 (2.02-2.45) | 1.73 (1.56-1.93) | 1.44 (1.26-1.64) |
| CI: confidence interval; BSI: bloodstream infection; CoNS: coagulase-negative staphylococci; CVC: central venous catheter; CVC-S: central venous catheter associated sepsis; HAI: healthcare-associated infection; HAP: healthcare-associated pneumonia; HAS: healthcare-associated sepsis; NEC: necrotising enterocolitis; VAP: invasive mechanical ventilation-associated pneumonia | | | | |

# T

**
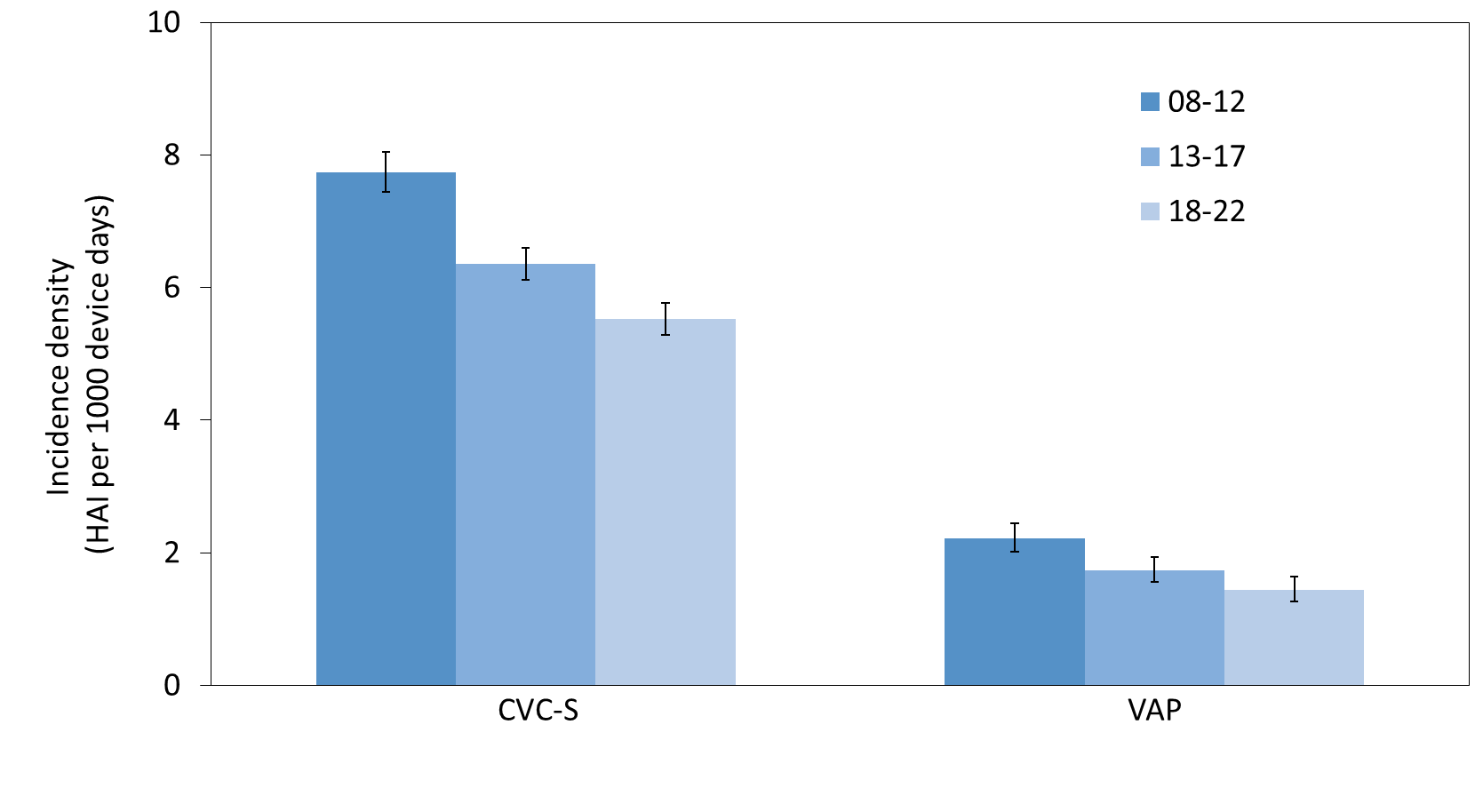
**

**Supplementary Figure S2: Incidence densities of device-associated healthcare-associated sepsis and pneumonia in neonates during the different surveillance periods, 2008-2012 vs. 2013-2017 vs. 2018-2022.**

CVC-S: central venous catheter-associated sepsis; HAI: healthcare-associated infection; VAP: invasive mechanical ventilation-associated pneumonia.

# **Supplementary Table S5: Descriptive data of neonates and acquired healthcare-associated infections for different gestational ages and birth weights**

| **Parameter** | **Gestational age*** | | | | **Birth weight** | | |
| --- | --- | --- | --- | --- | --- | --- | --- |
|  | <27w | 27-28w | 29-30w | >30w | <500g | 500-999g | 1000-1499g |
| Patients | 30952 | 26169 | 30697 | 29935 | 5041 | 44111 | 69062 |
| Patients died | 5290 | 981 | 530 | 398 | 1734 | 4319 | 1177 |
| Patient days | 1581773 | 1041125 | 908431 | 661037 | 270850 | 2122315 | 1813369 |
| CVC Days | 566270 | 257830 | 183688 | 89485 | 110343 | 650179 | 341068 |
| CVC days per 100 patient days (pooled mean) | 35.8 | 24.8 | 20.2 | 13.5 | 40.7 | 30.6 | 18.8 |
| Intubation Days | 389604 | 87024 | 42947 | 18362 | 85841 | 364927 | 89392 |
| Intubation days per 100 patient days (pooled mean) | 24.6 | 8.4 | 4.7 | 2.8 | 31.7 | 17.2 | 4.9 |
| Length of stay (days, pooled mean) | 51.1 | 39.8 | 29.6 | 22.1 | 53.7 | 48.1 | 26.3 |
| Mortality during surveillance per 100 patient days (95%CI) | 17.09 (16.49-17.52) | 3.75 (3.43-3.99) | 1.73 (1.53-1.8) | 1.33 (1.15-1.47) | 34.4 (32.53-35.73) | 9.79 (9.4-10.07) | 1.7 (1.57-1.8) |
| **Episodes** | | | | | | | |
| **HAS** | 8404 | 3340 | 2159 | 1308 | 1846 | 9222 | 4186 |
| CoNS BSI | 1884 | 843 | 646 | 389 | 388 | 2129 | 1254 |
| Non-CoNS BSI | 1968 | 774 | 542 | 366 | 362 | 2171 | 1123 |
| Clinical HAS | 4552 | 1723 | 971 | 553 | 1096 | 4922 | 1809 |
| CVC-S | 4569 | 1431 | 759 | 360 | 1069 | 4705 | 1363 |
| CVC-CoNS BSI | 1377 | 460 | 287 | 120 | 310 | 1453 | 488 |
| CVC-non-CoNS BSI | 1099 | 312 | 177 | 81 | 212 | 1120 | 338 |
| Clinical CVC-S | 2093 | 659 | 295 | 159 | 547 | 2132 | 537 |
| **HAP** | 1220 | 275 | 107 | 47 | 247 | 1210 | 200 |
| VAP | 793 | 126 | 38 | 22 | 187 | 714 | 83 |
| **NEC** | 1702 | 531 | 324 | 215 | 295 | 1812 | 679 |
| **Patients** | | | | | | | |
| **HAS** | 7114 | 3095 | 2087 | 1284 | 1479 | 8064 | 4076 |
| CoNS BSI | 1804 | 835 | 642 | 389 | 363 | 2063 | 1252 |
| Non-CoNS BSI | 1876 | 756 | 539 | 364 | 344 | 2082 | 1115 |
| Clinical HAS | 3965 | 1611 | 937 | 541 | 909 | 4400 | 1771 |
| CVC-S | 4180 | 1367 | 746 | 352 | 945 | 4373 | 1343 |
| **HAP** | 1175 | 269 | 103 | 46 | 232 | 1172 | 197 |
| VAP | 767 | 122 | 36 | 21 | 173 | 695 | 83 |
| **NEC** | 1686 | 526 | 323 | 214 | 290 | 1799 | 674 |
| **Incidence densities per 1000 patient days (95%CI)** | | | | | | | |
| HAS | 5.31 (5.2-5.43) | 3.21 (3.1-3.32) | 2.38 (2.28-2.48) | 1.98 (1.87-2.09) | 6.82 (6.51-7.13) | 4.35 (4.26-4.43) | 2.31 (2.24-2.38) |
| Non-CoNS BSI | 1.24 (1.19-1.3) | 0.74 (0.69-0.8) | 0.6 (0.55-0.65) | 0.55 (0.5-0.61) | 1.34 (1.2-1.48) | 1.02 (0.98-1.07) | 0.62 (0.58-0.66) |
| CoNS BSI | 1.19 (1.14-1.25) | 0.81 (0.76-0.87) | 0.71 (0.66-0.77) | 0.59 (0.53-0.65) | 1.43 (1.29-1.58) | 1 (0.96-1.05) | 0.69 (0.65-0.73) |
| Clinical HAS | 2.88 (2.79-2.96) | 1.65 (1.58-1.73) | 1.07 (1-1.14) | 0.84 (0.77-0.91) | 4.05 (3.81-4.29) | 2.32 (2.25-2.38) | 1 (0.95-1.04) |
| HAP | 0.77 (0.73-0.82) | 0.26 (0.23-0.3) | 0.12 (0.1-0.14) | 0.07 (0.05-0.09) | 0.91 (0.8-1.03) | 0.57 (0.54-0.6) | 0.11 (0.1-0.13) |
| NEC | 1.08 (1.03-1.13) | 0.51 (0.47-0.56) | 0.36 (0.32-0.4) | 0.33 (0.28-0.37) | 1.09 (0.97-1.22) | 0.85 (0.81-0.89) | 0.37 (0.35-0.4) |
| **Device-associated incidence densities per 1000 device days (95%CI)** | | | | | | | |
| CVC-S | 8.07 (7.84-8.31) | 5.55 (5.27-5.85) | 4.13 (3.84-4.44) | 4.02 (3.62-4.46) | 9.69 (9.12-10.29) | 7.24 (7.03-7.45) | 4 (3.79-4.21) |
| CVC-non-CoNS BSI | 1.94 (1.83-2.06) | 1.21 (1.08-1.35) | 0.96 (0.83-1.12) | 0.91 (0.72-1.13) | 1.92 (1.67-2.2) | 1.72 (1.62-1.83) | 0.99 (0.89-1.1) |
| CVC-CoNS BSI | 2.43 (2.3-2.56) | 1.78 (1.62-1.95) | 1.56 (1.39-1.75) | 1.34 (1.11-1.6) | 2.81 (2.51-3.14) | 2.23 (2.12-2.35) | 1.43 (1.31-1.56) |
| Clinical CVC-S | 3.7 (3.54-3.86) | 2.56 (2.36-2.76) | 1.61 (1.43-1.8) | 1.78 (1.51-2.08) | 4.96 (4.55-5.39) | 3.28 (3.14-3.42) | 1.57 (1.44-1.71) |
| VAP | 2.04 (1.9-2.18) | 1.45 (1.21-1.72) | 0.88 (0.63-1.21) | 1.2 (0.75-1.81) | 2.18 (1.88-2.51) | 1.96 (1.82-2.11) | 0.93 (0.74-1.15) |
| *missing value for gestational age in 461 cases. CI: confidence interval; BSI: bloodstream infection; CoNS: coagulase-negative staphylococci; CVC: central venous catheter; CVC-S: central venous catheter associated sepsis; HAI: healthcare-associated infection; HAP: healthcare-associated pneumonia; HAS: healthcare-associated sepsis; NEC: necrotising enterocolitis; VAP: invasive mechanical ventilation-associated pneumonia | | | | | | | |

# **Supplementary Table S6: Adjusted Hazard ratios for the different types of healthcare-associated infections in neonates calculated for the three surveillance periods by multivariable Cox-proportional hazard regression models.**

|  | **Type of healthcare-associated infection** | | | | | | | | | | | | | | |
| --- | --- | --- | --- | --- | --- | --- | --- | --- | --- | --- | --- | --- | --- | --- | --- |
|  | HAS | | | CVC-S | | | HAP | | | VAP | | | NEC | | |
|  | HR | 95%CI | p-value | HR | 95%CI | p-value | HR | 95%CI | p-value | HR | 95%CI | p-value | HR | 95%CI | p-value |
| Period 13-17 vs. 08-12 | 0.76 | 0.73-0.79 | <0.001 | 0.81 | 0.77-0.86 | <0.001 | 0.57 | 0.51-0.64 | <0.001 | 0.61 | 0.52-0.71 | <0.001 | 0.76 | 0.69-0.84 | <0.001 |
| Period 18-22 vs. 08-12 | 0.63 | 0.61-0.66 | <0.001 | 0.71 | 0.67-0.75 | <0.001 | 0.46 | 0.4-0.52 | <0.001 | 0.42 | 0.35-0.5 | <0.001 | 0.82 | 0.75-0.9 | <0.001 |
| Adjusted by: gender (female/male), birth weight (<500g/500-749g/750-999g/ 1000-1249g/1250-1499g), gestational age (<27w/27-28w/29-30w/>30w), delivery mode (Caesarean section yes/no), time of admission (</≥24h), multiple birth (yes/no), birth season (spring/summer/autumn/winter), patients per year (>30/≤30) and cluster effect of department | | | | | | | | | | | | | | | |
| CI: confidence interval; CVC-S: central venous catheter associated sepsis; HAP: healthcare-associated pneumonia; HAS: healthcare-associated sepsis; HR: Hazard ratio; NEC: necrotising enterocolitis; VAP: invasive mechanical ventilation-associated pneumonia | | | | | | | | | | | | | | | |

| **Parameter** | **Category** | **Type of HAS** | | | | | | | | | | | |
| --- | --- | --- | --- | --- | --- | --- | --- | --- | --- | --- | --- | --- | --- |
|  |  | Non-CoNS BSI | | | CoNS BSI | | | Clinical HAS | | | CVC-S | | |
|  |  | HR | 95%CI | p-value | HR | 95%CI | p-value | HR | 95%CI | p-value | HR | 95%CI | p-value |
| Period  vs 08-12 | 13-17 | 0.74 | 0.68-0.8 | <0.001 | 0.83 | 0.77-0.9 | <0.001 | 0.73 | 0.69-0.77 | <0.001 | 0.81 | 0.77-0.86 | <0.001 |
|  | 18-22 | 0.80 | 0.74-0.87 | <0.001 | 0.63 | 0.57-0.68 | <0.001 | 0.55 | 0.52-0.59 | <0.001 | 0.71 | 0.67-0.75 | <0.001 |
| Gender | female vs male | 0.86 | 0.8-0.92 | <0.001 | 0.83 | 0.78-0.89 | <0.001 | 0.76 | 0.73-0.8 | <0.001 | 0.82 | 0.78-0.86 | <0.001 |
| Birth weight  vs 1250-1499 g | <500 g | 1.56 | 1.29-1.89 | <0.001 | 1.99 | 1.65-2.39 | <0.001 | 3.03 | 2.65-3.47 | <0.001 | 5.36 | 4.66-6.17 | <0.001 |
|  | 500-749 g | 1.69 | 1.46-1.97 | <0.001 | 2.00 | 1.73-2.31 | <0.001 | 2.79 | 2.49-3.12 | <0.001 | 3.93 | 3.47-4.46 | <0.001 |
|  | 750-999 g | 1.14 | 0.99-1.31 | 0.06 | 1.34 | 1.17-1.52 | <0.001 | 1.77 | 1.59-1.97 | <0.001 | 2.29 | 2.03-2.59 | <0.001 |
|  | 1000-1249 g | 0.94 | 0.83-1.07 | 0.365 | 1.17 | 1.04-1.32 | <0.001 | 1.23 | 1.11-1.36 | <0.001 | 1.45 | 1.29-1.63 | <0.001 |
| Gestational age  vs >30 weeks | <27 weeks | 2.32 | 1.98-2.73 | <0.001 | 2.17 | 1.86-2.53 | <0.001 | 2.05 | 1.81-2.31 | <0.001 | 3.30 | 2.87-3.78 | <0.001 |
|  | 27-28 weeks | 1.51 | 1.31-1.76 | <0.001 | 1.54 | 1.34-1.77 | <0.001 | 1.56 | 1.39-1.75 | <0.001 | 2.03 | 1.78-2.32 | <0.001 |
|  | 29-30 weeks | 1.18 | 1.02-1.35 | 0.022 | 1.32 | 1.15-1.5 | <0.001 | 1.20 | 1.08-1.34 | 0.001 | 1.45 | 1.28-1.66 | <0.001 |
| Delivery mode  Vs vaginal | C. section | 0.73 | 0.66-0.81 | <0.001 | 1.11 | 0.99-1.24 | 0.087 | 0.94 | 0.87-1.02 | 0.158 | 0.89 | 0.82-0.96 | 0.003 |
| Admission  Vs inhouse birth | <24h after birth | 0.96 | 0.78-1.17 | 0.682 | 1.16 | 0.96-1.41 | 0.134 | 0.92 | 0.79-1.07 | 0.283 | 1.17 | 1.01-1.35 | 0.033 |
|  | >24h after birth | 0.64 | 0.51-0.8 | <0.001 | 0.69 | 0.56-0.85 | <0.001 | 0.79 | 0.68-0.91 | 0.001 | 0.70 | 0.61-0.81 | <0.001 |
| Multiple birth | yes vs no | 1.01 | 0.95-1.08 | 0.68 | 1.01 | 0.95-1.07 | 0.717 | 1.00 | 0.96-1.05 | 0.981 | 0.97 | 0.92-1.01 | 0.131 |
| Birth season (months)  vs 12-2 (winter) | 3-5 (spring) | 0.89 | 0.8-0.98 | 0.017 | 0.98 | 0.9-1.08 | 0.726 | 1.06 | 0.99-1.14 | 0.09 | 1.04 | 0.97-1.12 | 0.258 |
|  | 6-8 (summer) | 1.06 | 0.96-1.16 | 0.237 | 0.97 | 0.88-1.06 | 0.505 | 1.08 | 1.01-1.16 | 0.021 | 1.01 | 0.95-1.09 | 0.695 |
|  | 9-11 (autumn) | 1.05 | 0.96-1.16 | 0.282 | 1.00 | 0.91-1.1 | 0.954 | 1.02 | 0.95-1.1 | 0.542 | 1.07 | 1-1.15 | 0.046 |
| Enrolled infants per year | >30 vs <=30 | 0.95 | 0.84-1.07 | 0.362 | 1.17 | 1.01-1.34 | 0.035 | 0.97 | 0.87-1.07 | 0.519 | 0.93 | 0.84-1.03 | 0.184 |
| Model adjusted by cluster effect department. CI: confidence interval; BSI: bloodstream infection; CoNS: coagulase-negative staphylococci; CVC-S: central venous catheter associated sepsis; HAS: healthcare-associated sepsis; HR: Hazard ratio | | | | | | | | | | | | | |

# **Supplementary Table S7: Results of multivariable Cox-proportional Hazard regression analysis for the subtypes of healthcare-associated sepsis in neonates (adjusted full model)**
